# AAV Vector-Mediated Overexpression of CB1 Cannabinoid Receptor in Pyramidal Neurons of the Hippocampus Protects against Seizure-Induced Excitoxicity

**DOI:** 10.1371/journal.pone.0015707

**Published:** 2010-12-21

**Authors:** Stephan Guggenhuber, Krisztina Monory, Beat Lutz, Matthias Klugmann

**Affiliations:** Institute of Physiological Chemistry, University Medical Center of the Johannes Gutenberg University, Mainz, Germany; University of Chicago, United States of America

## Abstract

The CB1 cannabinoid receptor is the most abundant G-protein coupled receptor in the brain and a key regulator of neuronal excitability. There is strong evidence that CB1 receptor on glutamatergic hippocampal neurons is beneficial to alleviate epileptiform seizures in mouse and man. Therefore, we hypothesized that experimentally increased CB1 gene dosage in principal neurons would have therapeutic effects in kainic acid (KA)-induced hippocampal pathogenesis. Here, we show that virus-mediated conditional overexpression of CB1 receptor in pyramidal and mossy cells of the hippocampus is neuroprotective and moderates convulsions in the acute KA seizure model in mice. We introduce a recombinant adeno-associated virus (AAV) genome with a short stop element flanked by loxP sites, for highly efficient attenuation of transgene expression on the transcriptional level. The presence of Cre-recombinase is strictly necessary for expression of reporter proteins or CB1 receptor *in vitro* and *in vivo*. Transgenic CB1 receptor immunoreactivity is targeted to glutamatergic neurons after stereotaxic delivery of AAV to the dorsal hippocampus of the driver mice NEX-cre. Increased CB1 receptor protein levels in hippocampal lysates of AAV-treated Cre-mice is paralleled by enhanced cannabinoid-induced G-protein activation. KA-induced seizure severity and mortality is reduced in CB1 receptor overexpressors compared with AAV-treated control animals. Neuronal damage in the hippocampal CA3 field is specifically absent from AAV-treated Cre-transgenics, but evident throughout cortical areas of both treatment groups. Our data provide further evidence for a role of increased CB1 signaling in pyramidal hippocampal neurons as a safeguard against the adverse effects of excessive excitatory network activity.

## Introduction

Maintaining an optimal balance between excitatory and inhibitory activity of central nervous system (CNS) neurons is essential for proper physiological network activities, since either excessive glutamatergic transmission or insufficient GABAergic transmission can lead to excitotoxicity and epileptiform seizures in rodents and man [1]. The endocannabinoid system represents a molecular safeguard for efficient control of dangerous neuronal overexcitation [2], [3], [4]. Endocannabinoids are produced on-demand from endogenous lipid precursors, and act as retrograde messengers that inhibit release of neurotransmitters by the activation of presynaptic cannabinoid type 1 (CB1) receptors. CB1 receptor activation has anticonvulsant and neuroprotective effects in acute and chronic seizure models [5], [6] and extracts of the plant Cannabis sativa have been used as epilepsy medication since thousands of years [4]. CB1 is expressed on both GABAergic interneurons and glutamatergic principal neurons in the hippocampus [2], [7], [8], [9], [10], a region strongly implicated in the development of epilepsy. The endocannabinoid system has been implied as a therapeutical target in epilepsy [11] and as such, effective treatment strategies utilizing CB1 receptor regulation require a detailed understanding of CB1 receptor effects in neuronal subtypes. To this end, the analysis of conditional mouse mutants lacking CB1 receptors on different subtypes of neurons subjected to the kainic acid (KA)-induced seizures revealed that CB1 receptors on hippocampal glutamatergic but not GABAergic neurons are required for protection against excitotoxic seizures [10]. In line with this pre-clinical data, specific down-regulation of protein and mRNA of CB1 receptor on glutamatergic, but not on GABAergic axon terminals had been reported in epileptic human hippocampal tissue [12]. However, these conditional loss-of-function studies have not yet been complemented by the corresponding gain-of function approach entailing CB1 overexpression, preventing a comprehensive picture of CB1-mediated control of overexcitation.

Here we examined the effects of increased CB1 gene dosage in the hippocampus on the development of epileptiform seizures and neuronal damage in the KA model. To that end, we have used adeno-associated virus (AAV) vectors for the delivery of the CB1 receptor to the hippocampus of adult mice. Because of its inherent neurotropism, stereotaxic delivery of AAV has been used widely for gene transfer to the rodent hippocampus in the context of animal models of excitotoxic seizures [10], [13]. However, conventional AAV vectors transduce all types of hippocampal neurons [14] that might result in confounding results. To avoid this confounding factor, we restrict transgene expression exclusively to principal neurons expressing Cre-recombinase, using an AAV-expression cassette with a transcriptional stop cassette flanked by loxP sites, preceding the transgene. In this study, we show that somatic transfer of the CB1 gene to glutamatergic hippocampal neurons is sufficient to provide protection against acute seizures and neuronal damage.

## Results

### AAV-Stop-mediated transgene expression requires Cre-induced recombination

**Figure 1:**
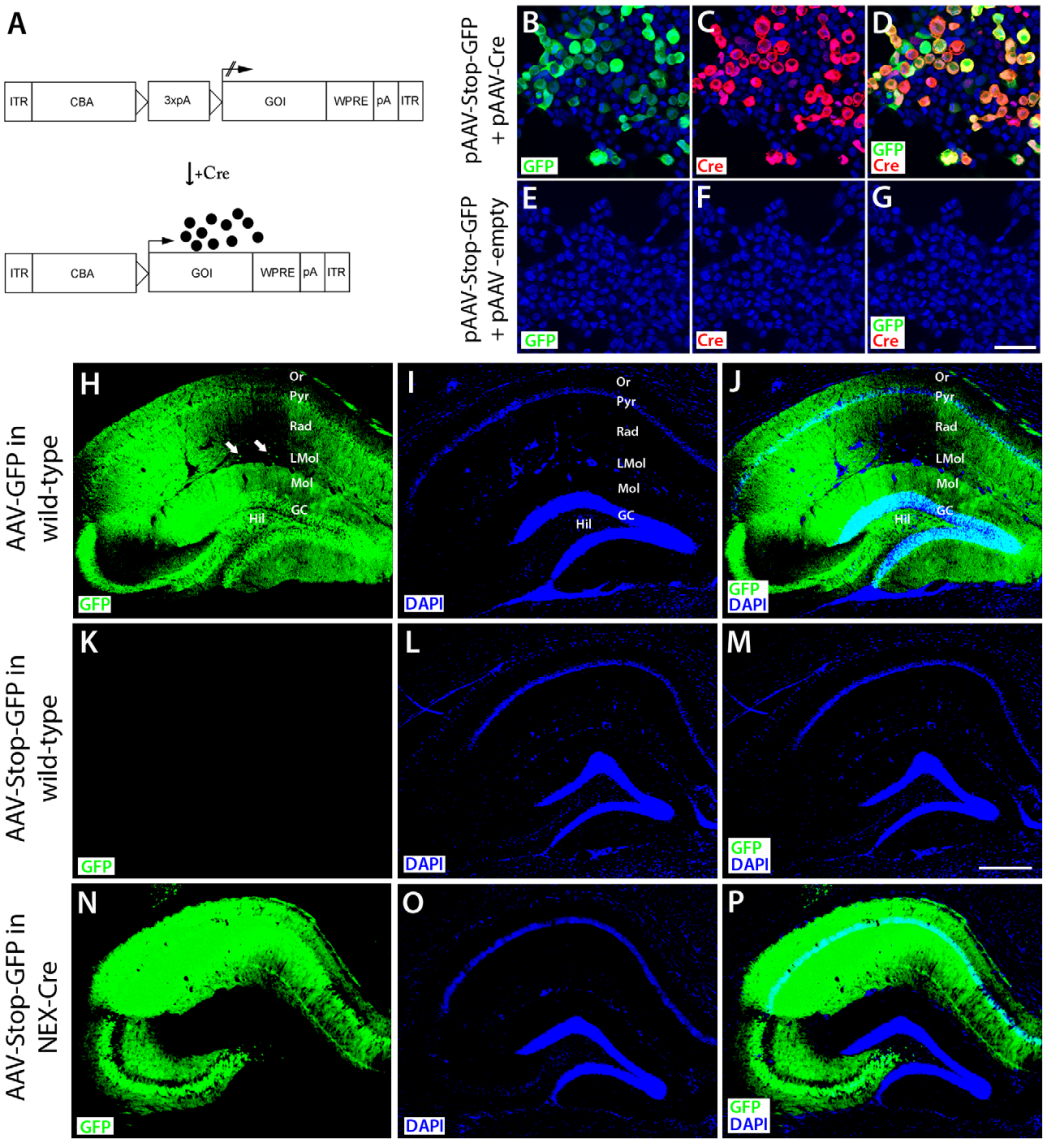
Cre recombinase-activated transgene expression. **A**, AAV expression cassette. Top, Silencing of transgene expression by transcriptional termination cassette containing three polyadenylation sites flanked by loxP sites (triangles). Bottom, Cre recombinase-mediated excision of the Stop cassette leading to transcription of the transgene. ITR, inverted terminal repeat; CBA, chicken-β-actin promoter; 3xpA, three polyadenylation (pA) signals; GOI, gene of interest; WPRE, woodchuck hepatitis virus post-transcriptional regulatory element. **B–G**, HEK cells were co-transfected with pAAV-Stop-GFP and pAAV-Cre (B–D) or pAAV-empty (E–G) and GFP immunofluorescence is strictly limited to Cre transfectants. Transgene expression is tightly inhibited when pAAV-Stop-GFP is co-transfected with pAAV-empty. Blue: cell nucleus staining with DAPI, Green: GFP immunostaining, Red: Cre recombinase immunostaining. Bar in G: 50 µm. **H–P**, Two months after stereotaxic vector delivery (AAV-GFP or AAV-Stop-GFP) to the dorsal hippocampus of adult wild-type or NEX-Cre mice, GFP epifluorescence was assessed in brain sections. **H–J**, AAV-GFP efficiently transduces all types of neurons of the hippocampal formation, in particular in CA1, CA2, CA3, the hilar region and the dentate gyrus. Note that transduced interneurons (arrowheads in H) can be visualized in areas of low GFP abundance. GC, granule cell layer; Hil, hilar region; LMol, stratum lacunosum-molecularis; Mol, stratum molecularis; Or, stratum oriens; Pyr, CA1/CA3 pyramidal cell layer; Rad, stratum radiatum. **K–M**, After AAV-Stop-GFP injection, GFP expression was not detectable in wild-type mice. **N–P**, In NEX-Cre mice, neurons of the pyramidal cell layer express the reporter gene, while granule cells of the dentate gyrus are spared. Note that in this mouse line, Cre recombinase is not expressed in the adult dentate gyrus (see Goebbels et al., 2006). Bar in M: 250 µm.

The neurotropic AAV1/2 has previously been shown to efficiently deliver genes to all neuronal subtypes of the rodent hippocampus [10], [14]. To restrict virus-mediated transduction to glutamatergic hippocampal neurons our approach was to excise a transcriptional termination element preceding the cDNA in the AAV-expression cassette by providing Cre-recombinase in *trans* (Fig. 1A). The packaging limit of AAV is 5.2 kb [15] and accommodation of large transgenes requires minimizing the size of *cis* elements in the expression cassette. To adhere to that concept, we designed a transcriptional termination (‘Stop’) element entailing two loxP sites (34 bp) flanking a herpes simplex virus thymidin kinase polyadenylation site (70 bp) and a polyadenylation terminator (154 bp). The Stop cassette was cloned into our latest generation AAV expression cassette [13] between the cytomegalovirus enhancer/chicken beta actin (CBA) promoter and the hrGFP (humanized renilla green fluorescent protein) cDNA to obtain pAAV-Stop-GFP. We transfected human embryonic kidney (HEK 293) cells with this reporter construct to assess the efficacy of the Cre-induced AAV system *in vitro*. Immunocytochemical analysis showed that the absence of Cre recombinase prevented transcription of the reporter (Fig. 1E-G), whereas co-transfection with a Cre plasmid caused efficient activation of GFP expression (Fig. 1B–D).

**Figure 2:**
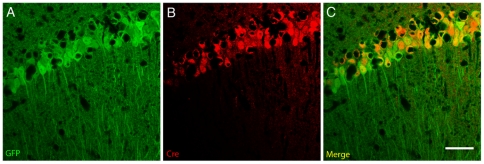
Recombination of AAV genomes is restricted to Cre-expressing neurons in vivo. AAV-Stop-GFP was injected to the hippocampus of NEX-Cre transgenic mice and GFP expression visualized in brain sections two months later. Transduced CA1 pyramidal neurons express GFP (A) and Cre recombinase (B). As expected, the merge picture (C) shows segregated subcellular expression domains of the cytosolic GFP and the nuclear Cre. Bar: 25 µm.

For *in vivo* analysis, we injected either generic AAV-GFP or conditional AAV-Stop-GFP to the hippocampus of adult (>2 months) wild-type mice (C57BL/6N) and Cre driver mice expressing Cre specifically in glutamatergic forebrain neurons under the control of regulatory sequences of the NEX gene (NEX-cre). The NEX gene is active in pyramidal neurons and dentate gyrus mossy cells, but not in interneurons, oligodendrocytes and astrocytes, nor in granule cells of the dentate gyrus after P10 [16]. Mice were sacrificed at 4 weeks post-injection when AAV-mediated transgene expression had peaked to remain at stable levels and vector spread was determined by immunohistochemistry. GFP immunoreactivity was observed throughout the dorsal hippocampus of AAV-GFP-injected mice. As expected, transduction of all types of neurons occurred in the hippocampal formation, CA1-3, hilar region and the dentate gyrus (Fig. 1H–J). The abundant reporter protein expression in processes of principal neurons generally prevented visualization of transduced interneurons. However, assessment of sections showing moderate GFP levels in stratum radiatum and stratum lacunosum unmasked the presence of GFP-immunoreactivity also in interneurons (Fig. 1H). In contrast, no GFP immunoreactivity was detected after delivery of AAV-Stop-GFP into wild-type mice (Fig. 1K–M), even after prolonged exposure times (not shown), validating the effective attenuation of transcription by the Stop cassette *in vivo*. In AAV-Stop-GFP treated NEX-Cre mice (AAV-Glu-GFP), pyramidal neurons showed robust GFP expression (Fig. 1N–P). As expected, dentate granule cells were unlabeled indicating the lack of Cre expression in these neurons at adult stages when AAV-delivery was performed [16]. Co-localisation of GFP and Cre immunoreactivity in the CA1 region of NEX-Cre mice suggests that activation of transgene expression depends on the Cre-mediated recombination of AAV genomes (Fig. 2). In summary, these findings demonstrate that the presence of a small stop element confers tight spatio-temporal control over transgene expression after AAV delivery to Cre driver mouse lines.

### Conditionally expressed CB1 receptor in hippocampal pyramidal cells is functional

**Figure 3:**
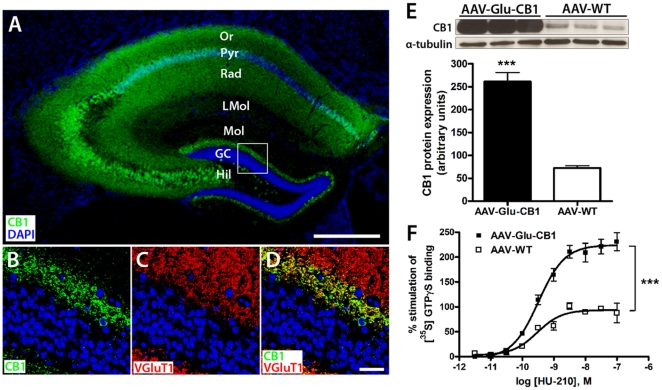
Transgenic HA-tagged CB1 receptor is expressed in hippocampal pyramidal neurons. AAV-Stop-CB1 was injected bilaterally to the hippocampus of NEX-Cre transgenic (AAV-Glu-CB1) mice and their respective wild-type (AAV-WT) littermates. **A**, Cre-activated CB1 expression occurred in pyramidal neurons and appeared in a similar pattern compared to AAV-Stop-GFP confirming the integrity of the system. GC, granule cell layer; Hil, hilar region of dentate gyrus; LMol, stratum lacunosum-molecularis; Mol, stratum molecularis; Or, stratum oriens; Pyr, CA1/CA3 pyramidal cell layer; Rad, stratum radiatum. Bar: 250 µm. **B-D**, Higher magnification micrographs of dentate granule cells shown in A. Immunohistochemistry for the HA-tag reveals co-expression of CB1 and VGluT1 in the inner molecular layer of the dentate gyrus, validating the presynaptic localization of transgenic CB1 receptor. Bar in D: 25 µm. **E**, Western blot analysis of hippocampal homogenates of AAV-Glu-CB1 (n = 3) and AAV-WT (n = 3) mice. AAV-Glu-CB1 mice express significantly increased levels of CB1 receptor protein. Data are normalized for α-tubulin (p<0.001; unpaired t test analysis, two-tailed). **F**, Stimulation of [^35^S]GTPγS binding in hippocampal homogenates of AAV-Glu-CB1 mice (n = 6) and AAV-WT littermates (n = 3) was determined by various concentrations of the CB1 agonist HU-210. Basal binding was measured in absence of HU-210 and defined as 0% in each experiment. Data are presented as percentage stimulation above basal [^35^S]GTPγS binding and are the means ± SEM, all performed in duplicates. The non-linear regression curve illustrates that overexpression of the CB1 receptor in hippocampal pyramidal neurons resulted in significantly enhanced cannabinoids-induced G-protein activation and thus increased cannabinoid signaling. EC_50_ = 3.26±0.08 nM (AAV-Glu-CB1), 2.97±0.13 nM (AAV-WT); E_max_  = 224.4±7.39 (AAV-Glu-CB1), 93.77±4.99 (AAV-WT). Unpaired t test analysis, two-tailed: p<0.0001.

CB1 receptor is known to be expressed in distinct neuronal subpopulations in the hippocampus [9], [10], [17] with very high levels in GABAergic interneurons belonging mainly to the cholecystokinin-positive and parvalbumin-negative type [9], [18] and 20–30 times less CB1 receptor protein in glutamatergic pyramidal terminals [17], [19]. For a cell-type specific CB1 overexpression, we replaced the reporter in pAAV-Stop-GFP with the coding region of the HA-tagged rat CB1 receptor cDNA. The presence of the HA-epitope tag facilitates immunological detection of the transgene and allows specific assessment of ectopic versus endogenous CB1 receptor protein. The AAV-Stop-CB1 vector was administered to the hippocampus of adult NEX-Cre mice to achieve conditional CB1 overexpression in hippocampal glutamatergic cells (AAV-Glu-CB1 mice). Wild-type littermates of NEX-Cre mice that do not express Cre recombinase were also injected with the AAV-Stop-CB1 vector and served as control group (named as AAV-WT mice). Immunohistochemical detection of the HA-tag revealed Cre-activated CB1 receptor expression in hippocampal pyramidal neurons (Fig. 3A) in a similar pattern compared to AAV-Stop-GFP-injected animals confirming the integrity of the conditional AAV system. Co-localisation of ectopic CB1 receptor and VGluT1 (vesicular glutamate transporter 1), a marker for glutamatergic presynaptic sites, in the inner third of the molecular layer of the dentate gyrus where the mossy cells are synapsing on granule cell dendrites demonstrated presynaptic location of ectopic CB1 protein (Fig. 3B–D). The subcellular localization in glutamatergic cells of exogenous CB1 receptor protein matches that of the endogenous receptor [10] and the detection of HA-immunoreactivity in somata is likely to reflect accumulated CB1 receptors destined for transport to axonal terminals.

Furthermore, we examined CB1 protein levels in hippocampal homogenates of AAV-Glu-CB1 and AAV-WT mice that were sacrificed four weeks post-injection by western blot analysis (Fig. 3E). CB1 receptor protein levels were significantly increased in AAV-Glu-CB1 mice compared to controls following normalization to tubulin (p<0.001; unpaired t test, two tailed). Increased CB1 receptor levels may result in enhanced cannabinoid-induced G protein activation and thus enhanced endocannabinoid signaling. To address that, we performed HU-210-stimulated [^35^S]GTPγS binding with the same hippocampal homogenates of AAV-Glu-CB1 and AAV-WT mice used for immunoblot analysis. AAV-WT mice reached a maximum stimulation of 93.8%, over baseline, while HU-210-induced G protein activation was significantly increased in AAV-Glu-CB1 mice, reaching a maximum of 224.4% (Fig. 3F; p = 0.0001, unpaired t test, two-tailed). Our quantitative biochemical analyses revealed an overall CB1 receptor upregulation of 2.5-fold compared with controls. However, this value might underestimates the true increase of CB1 receptor in AAV-Stop-CB1 treated pyramidal neurons, given the moderate expression levels of endogenous CB1 protein in this neuronal subtype.

Taken together, we demonstrated ectopic CB1 receptor to be robustly expressed cell-type specifically in hippocampal pyramidal neurons, to be located at presynaptic sites and to be coupled to G proteins. Thus, ectopic CB1 receptor showed common characteristics of endogenous CB1 protein.

### Increased CB1 receptor gene dosage in hippocampal glutamatergic neurons confers protection against epileptiform seizures

**Figure 4:**
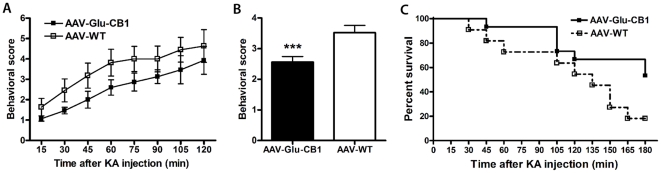
Behavioural effects of elevated CB1 receptor levels in hippocampal pyramidal neurons and mossy cells. Seizures were induced in AAV-Glu-CB1 mice (n = 15) and AAV-WT controls (n = 11) by i.p. injection of kainic acid (30 mg/kg). **A**, Seizure severity was reduced in AAV-Glu-CB1 mice at every time point of scoring compared to AAV-WT controls without reaching significance (p = 0.065, Mann Whitney test, two-tailed). **B**, The average behavioral score over a period of 120 min is significantly decreased in AAV-Glu-CB1 mice (p = 0.0007, Mann Whitney test, two-tailed) indicating improved protection against KA-induced seizures. **C**, Kaplan–Meier survival curves of AAV-Glu-CB1 and AAV-WT mice during KA treatment are significantly different between both genotypes (p = 0.0494, log rank test). The survival rate at 180 min after KA injection was 53% of AAV-Glu-CB1 vs. 18% of AAV-WT mice.

CB1 activation on glutamatergic neurons has been shown to play an essential role in the protection against excitotoxic seizures [5], [6], [10], [20]. This provided the rationale to investigate the therapeutic potential of CB1 receptor overexpression in the context of the pathogenic consequences of experimentally induced overexcitation of glutamatergic circuits in the hippocampus. Kainic acid (KA) was injected (30 mg/kg, i.p.) to AAV-Glu-CB1 and AAV-WT mice to induce robust activation of excitatory pathways resulting in acute epileptiform seizures. At every time point of scoring seizure severity was moderated in AAV-Glu-CB1 mice compared to wild-type controls (Fig. 4A) without reaching statistical significance (p = 0.065, Mann Whitney test, two-tailed). However, the average behavioral score over a period of 120 min is significantly decreased in AAV-Glu-CB1 mice (Fig. 4B, p = 0.0007, Mann Whitney test, two-tailed). Severe KA-induced motor seizures can be fatal. Kaplan-Meier survival analysis demonstrated a significant difference between both genotypes in the course of the KA treatment (Fig. 4C, p = 0.0494, log rank test). At 60 min after KA-injection, 93% of AAV-Glu-CB1 mice (n = 15) versus 72% of control animals (n = 11) were alive (Fig. 4C). 180 min after the start of the experiment, 53% of AAV-Glu-CB1 mice but only 18% of AAV-WT mice had survived. No animals died at later stages.

Importantly, we have shown previously, that expression of Cre recombinase in glutamatergic neurons of the forebrain *per se* does not alter susceptibility to KA-induced seizures [5]. These results show that CB1 overexpression in glutamatergic hippocampal neurons ameliorates the severity of acute epileptiform seizures indicating an essential role of hippocampal pyramidal neurons and mossy cells in CB1-dependent on-demand protection against excessive excitatory activity.

### CB1 receptor overexpression and excitotoxicity

**Figure 5:**
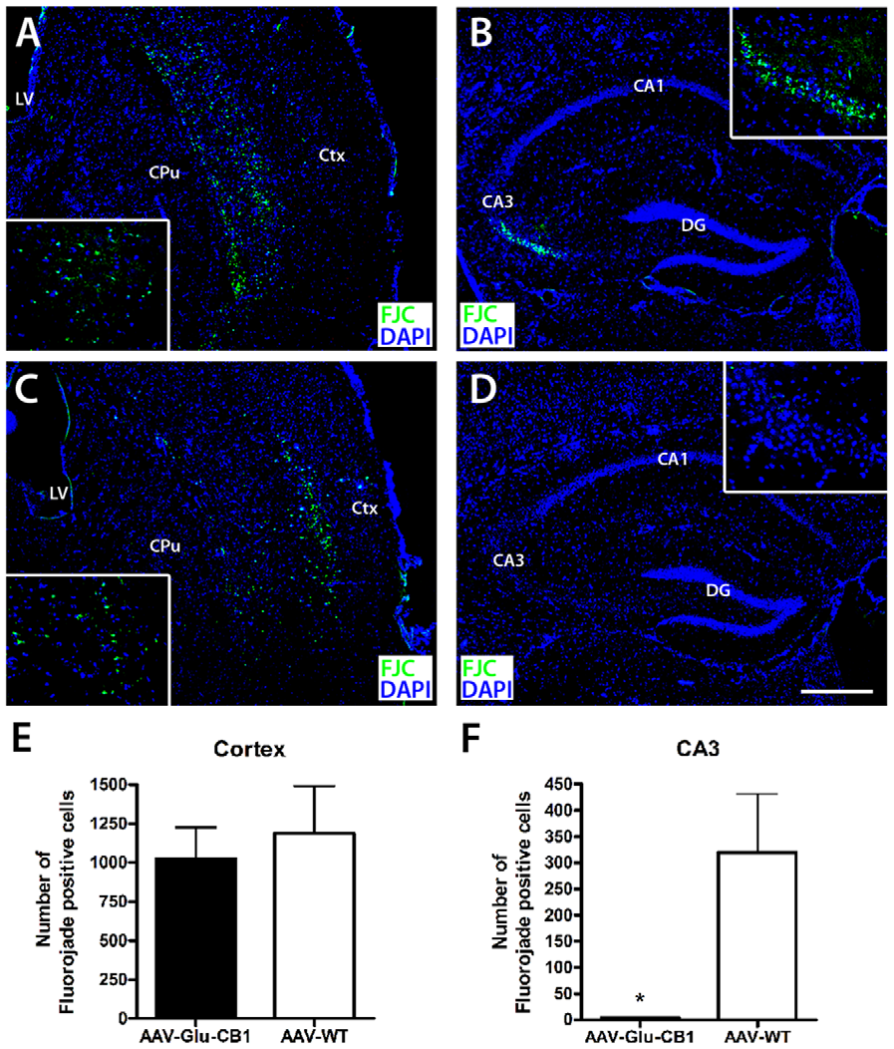
Increased CB1 receptor levels prevent degeneration of CA3 pyramidal neurons. Assessment of neurodegeneration by Fluoro-Jade C (FJC) staining 5 days after exposure to KA. **A–D**, Representative images show FJC staining in the cortex and hippocampus of AAV-WT mice (A, B) and AAV-Glu-CB1 mice (C, D). Insets show magnifications of FJC-labeled neurons in the cortex (A, C) and the CA3 area (B, D). CPu, caudate putamen; Ctx, cortex; DG, dentate gyrus; LV, lateral ventricle. Bar in D: 250 µm. **E**, Quantification of FJC-labeled neurons demonstrated comparable levels of neurodegeneration in the cortex in both groups. **F**, In contrast, degeneration of CA3 pyramidal neurons was blocked in AAV-Glu-CB1 but not in AAV-WT mice (p<0.05, unpaired t test analysis, two-tailed).

Systemic KA treatment leads to neuronal degeneration especially in CA3 pyramidal neurons of the hippocampus [1]. Five days after kainic acid injections, mice were sacrificed and brain sections were stained with Fluoro-Jade C (FJC), a green fluorescent dye specific for labeling degenerating neurons [21]. Robust neuronal cell death was evident in subcortical areas of both AAV-Glu-CB1 (n = 6) and AAV-WT (n = 5) mice; however the hippocampus of AAV-Glu-CB1 was virtually spared. Representative pictures of FJC-labeled cells in the CA3 area (Fig. 5B,D) and in the neocortex (Fig. 5A,C) of AAV-Glu-CB1 and AAV-WT mice are shown. Fluoro-Jade labeling of degenerating neurons is known to be preserved even 2 weeks after KA injection [22], excluding the possibility that CA3 pyramidal cells of AAV-Glu-CB1 mice might have already died 5 days after KA injection and hence did not stain. Moreover, FJC-positive cell counts in the cortex, revealed a similar extent of neurodegeneration in both groups (Fig. 5E), but neuronal damage was almost absent in the CA3 area of AAV-Glu-CB1 mice (Fig. 5F, p = 0.012, unpaired t test analysis, two-tailed). This finding demonstrates that genetically increased levels of CB1 receptor in glutamatergic cells of the hippocampus is sufficient to provide protection from excitotoxic cell death after prolonged motor seizures.

## Discussion

Our data demonstrate that incorporation of the highly versatile Cre-loxP system [23] into the AAV platform allows tight control over expression of almost any gene in subsets of neurons under very precise spatio-temporal control *in vivo.* The Cre-activated AAV system affords a broad application spectrum comprising (1) analysis of the Cre expression pattern of newly generated cell-type specific Cre driver mice; (2) intense labelling of neurons to trace long-range axon projections to reveal connectivity of specific regions within the brain; (3) manipulation of neurons by expression of light-activated ion channels to selectively induce network activity [24], [25], [26] and (4) overexpression of the gene of interest in a neuronal subpopulation. Cre-activated transgene expression in a neuronal subpopulation is determined by the transgenic mouse line driving Cre recombinase under a cell-type specific promoter. To achieve an overexpression of the gene of interest, Cre expression must resemble the expression pattern of the gene of interest in the particular brain region. Therefore, the choice of the Cre driver line requires serious consideration. In our proof-of-principle experiments, analysis of Cre-mediated recombination revealed reporter gene expression in the NEX-Cre line in pyramidal CA1, CA2 and CA3, and hilar mossy cells while dentate granule cells are spared (Fig. 1N–P). Hence, Cre expression in the hippocampus of NEX-Cre mice resembles endogenous CB1 receptor expression in hippocampal glutamatergic neurons [10].

In the present study, we demonstrate that increased CB1 receptor levels in hippocampal glutamatergic neurons protect against excitotoxic seizures. This finding is in agreement with previous conditional knock-out studies that CB1 activation on glutamatergic terminals, especially of mossy cells projecting to dentate granule cells, plays an essential role in the protection against excitotoxic seizures [5], [10], while genetic CB1 ablation from GABAergic interneurons had no effect on seizure severity [10]. Excessive excitatory neurotransmission causes an increase in Ca^2+^ influx leading to neuronal degeneration, a harmful process known as excitotoxicity [1]. The dispersal of excessive excitatory neurotransmission finally results in behavioral symptoms, such as paroxysmal seizures. It is thought that this procedure participates in the progress of various forms of epilepsy. The endocannabinoid system can dampen glutamatergic transmission via CB1 receptors [2], [3], [19], [27] and therefore represents a control system to limit the danger of excessive excitatory activity. Our genetic data are supported by the findings of several pharmacological studies. Anti-convulsive effects in acute seizure models were demonstrated after increasing endocannabinoid signaling through systemic administration of CB1 receptor agonists [28], or endocannabinoid degradation inhibitors [5], [29], [30], while application of CB1 receptor antagonist SR141716A can block the anticonvulsant effect of cannabinoids [5], [28]. Moreover, seizure activity is accompanied by increased synthesis of the endocannabinoids anandamide [5] and 2-AG [6], [31].

In the hippocampus of patients with temporal lobe epilepsy CB1 receptor expression and the fraction of glutamatergic axon terminals equipped with CB1 are downregulated [12]. As a consequence, negative feedback control at excitatory synapses is impaired in epileptic patients. Interestingly, recent studies demonstrated that CB1 receptor expression undergoes a long-term redistribution in the hippocampus following epileptogenesis in the pilocarpine model of acquired epilepsy [6], [20]. Falenski et al. suggested that the redistribution might serve as a compensatory effect comprising an upregulation of CB1 receptor in glutamatergic and a downregulation in GABAergic neurons [20] and our results support the hypothesis that CB1 receptor in glutamatergic hippocampal neurons is essential to provide endogenous protection against KA-induced seizures. However, recent reports on an anti-convulsive role of CB1 receptor signaling in GABAergic neurons in different seizure models suggest that a potential therapeutic efficacy of cannabinoids might depend on the type of epilepsy [32], [33].

We demonstrated that elevated CB1 receptor levels safeguard from neuronal cell death caused by excessive excitatory neurotransmission. This result is consistent with our previous studies [5], [10], showing that genetic deletion of CB1 receptor from principal forebrain neurons results in higher levels of neuronal degeneration in the hippocampus following KA treatment.

In conclusion, we provided strong evidence for the protective role of CB1 receptor on hippocampal glutamatergic terminals as a molecular stout guard in controlling excessive network activity [4]. Thus, CB1 receptor expression on hippocampal glutamatergic neurons may represent a target for novel agents to restrain excitotoxic events and to treat neurodegenerative diseases.

## Materials and Methods

### Ethics statement

Experiments were approved by the local animal care committee (Landesuntersuchungsamt Koblenz, permit numbers 23177-07/051-47V1 and 23177/G10-1-037).

### DNA constructs

All constructs used in this study were based on a AAV expression cassette containing the 1.1 kb CMV immediate early enhancer/chicken β-actin hybrid promoter (CBA), the humanized renilla GFP (hrGFP) cDNA, the woodchuck hepatitis virus post-transcriptional regulatory element (WPRE), and the bovine growth hormone polyadenylation sequence (bGHpA) flanked by AAV2 inverted terminal repeats (pAAV-GFP). The 340 bp transcriptional Stop cassette was designed to entail a herpes simplex virus thymidin kinase pA signal and a pA terminator from pGL3 (Promega, Madison, USA) flanked by loxP sites, and was synthesised by a commercial provider (Epochbiolabs, Missouri City, USA). The Stop cassette was then transferred into the BamHI-site downstream of CBA in pAAV-GFP to obtain pAAV-CBA-Stop-GFP-WPRE-bGHpA (pAAV-Stop-GFP). The rat CB1 open reading frame, fused downstream the coding region of the hemagglutinin (HA)-tag, replaced GFP in pAAV-Stop-GFP to obtain pAAV-CBA-Stop-CB1-WPRE-bGHpA (pAAV-Stop-CB1). In addition, the pAAV backbone with no cDNA (pAAV-empty) or with the sequence encoding Cre-recombinase fused to the HA-tag and a nuclear localization signal (pAAV-Cre) were used for *in vitro* experiments.

### AAV vector administration

Production of pseudotyped AAV1/2 chimeric vectors and determination of genomic titres using the ABI 7500 real time PCR cycler (Applied Biosystems) were performed as described [13], [34]. Adult male NEX-Cre mice [16], and wild-type littermates (26–30 g) were anaesthetised as described [35], and 1 µl of either AAV-GFP, AAV-Stop-GFP or AAV-Stop-CB1 (7×10^10^ viral genomes/ml) was injected bilaterally into the hippocampus (+2.0 mm AP, ±2.0 mm ML, −2.0 mm DV from bregma). Vector delivery was performed at a rate of 150 nl/min using a microprocessor controlled mini-pump (World Precision Instruments, Sarasota, FA, USA) with 34xG beveled needles (World Precision Instruments) in a stereotaxic frame (Kopf Instruments, Tujunga, CA, USA).

### Immunohistochemistry

Brain sections were Immunostained as described [35]. Briefly, free-floating brain sections (40 µm) were incubated overnight with the following primary antibodies (1∶1000 in immunobuffer): rabbit anti-hrGFP (Stratagene, LaJolla, CA), rabbit anti-Cre (Covance, Richmond, CA), rabbit anti-HA (Santa Cruz Biotechnology, Santa Cruz, CA), and guinea pig anti-VGlut1 (Chemicon, Temecula, CA). Sections were washed and then incubated for 1 h with the appropriate Alexa488 or Alexa546-conjugated goat IgG (1∶1000, Invitrogen, Eugene, OR). Before the third wash in PBS, sections were counterstained with the nuclear dye 4′,6-diamidino-2-phenylindole (DAPI) for 5 min. Sections were then transferred onto glass slides and coverslipped with Moviol mounting medium and fluorescence was visualized using a Olympus SZ61 Stereo microscope (Olympus Corporation, Tokyo, Japan) or a laser scanning confocal microscope (Zeiss, Axiovert LSM 710).

### Induction of acute excitotoxic seizures

Kainic acid (Ascent scientific, Bristol, UK) was dissolved in 0.9% saline and administered (30 mg/kg; i.p.) in a volume of 10 ml/kg body weight to induce epileptiform seizures. A trained observer blind to the genotype of the mice monitored the severity of seizures for 2 hrs and scored every 15 min according to the following scale [10]: 0 - no response; 1 - immobility and staring; 2 - forelimb and/or tail extension, rigid posture; 3 - repetitive movements, head bobbing; 4 - rearing and falling; 5 - continuous rearing and falling: 6 - severe clonic-tonic seizures; 7 - death.

### Fluoro-Jade staining

For detection and quantification of neuronal degeneration evoked by kainic acid injection, Fluoro-Jade C (Millipore, Schwalbach, Germany) staining was performed as described previously [36]. Sections were collected every 120 µm for analysis of neuronal degeneration of hippocampal neurons in each mouse (10 sections per animal). Fluoro-Jade staining was visualized under a FITC filter and quantified by two independent observers unaware of the genotype.

### Western Blot

Hippocampi of AAV-Glu-CB1 mice and AAV-WT mice were isolated and homogenized in 1 ml of ice-cold membrane buffer (50 mM Tris-HCl, pH 7.4, 3 mM MgCl2, 0.2 mM EGTA, Complete protease inhibitor; Roche, Basel, Switzerland). Following determination of protein content, aliquots were mixed with an equal volume of 2× Laemmli reducing sample buffer. 10 µg of total protein were resolved by 12% SDS-PAGE and electro-blotted onto nitrocellulose membrane. After blocking in 5% non-fat dry milk, the membrane was incubated with primary antibodies (αCB1 (Frontier Sciences, Hokkaido, Japan) or rabbit αHA (at 1∶1000) at 4°C overnight. α-tubulin (Sigma-Aldrich, St. Louis, MO) was used as loading control. Secondary antibodies were horseradish peroxidase-conjugated anti-rabbit or anti-mouse IgG (Dianova, Hamburg, Germany). Western blot analysis was performed using a chemiluminescence system (Luminol). Detection was made by autoradiography.

### Agonist-stimulated [^35^S]GTPγS Binding

Briefly, hippocampus homogenates (10 µg) of AAV-Glu-CB1 mice and AAV-WT mice, in which AAV-mediated CB1 expression had been analyzed by immunoblot, were processed for the binding assay as described [37].

### Data analysis

Data are presented as means ± SEM and were analyzed using unpaired, two-tailed t-test for normally distributed variables to evaluate statistical significance with p<0.05 as level of statistical significance. Non-normally distributed seizure scores were analyzed using the two-sided Mann-Whitney U-test with p<0.05 as level of statistical significance. Western blot quantification was performed using NIH ImageJ (Image Processing and Analysis in Java). For agonist-stimulated [^35^S]GTPγS binding, data were analyzed with GraphPad Prism 4.0 software using nonlinear regression and sigmoidal curve fitting to obtain potency (EC_50_) and efficacy (E_max_) values. Basal binding was measured in the absence of cannabinoid receptor agonists and defined as 0% in each experiment. All data are expressed as percentage stimulation above basal [^35^S]GTPγS binding. The Kaplan-Meier method was used to evaluate survival, followed by the log rank test to identify significant differences. Graphs and statistics were generated by GraphPad Prism 4.0 (GraphPad Software, La Jolla, CA).
